# The promise of the anti-idiotype concept

**DOI:** 10.3389/fonc.2012.00196

**Published:** 2012-12-19

**Authors:** Thomas Kieber-Emmons, Bejatohlah Monzavi-Karbassi, Anastas Pashov, Somdutta Saha, Ramachandran Murali, Heinz Kohler

**Affiliations:** ^1^Winthrop P. Rockefeller Cancer Institute, Department of Pathology, University of Arkansas for Medical SciencesLittle Rock, AR, USA; ^2^Institute of Microbiology, Bulgarian Academy of SciencesSofia, Bulgaria; ^3^Department of Biomedical Sciences, Cedars Sinai Medical CenterLos Angeles, CA, USA; ^4^Department of Microbiology and Immunology, University of KentuckyLexington, KY, USA

**Keywords:** anti-idiotype, carbohydrate mimetic peptides, idiotype network theory, cancer, tumor, mimotopes, antibody, vaccine design

## Abstract

A basic tenet of antibody-based immunity is their specificity to antigenic determinates from foreign pathogen products to abnormal cellular components such as in cancer. However, an antibody has the potential to bind to more than one determinate, be it an antigen or another antibody. These observations led to the idiotype network theory (INT) to explain immune regulation, which has wax and waned in enthusiasm over the years. A truer measure of the impact of the INT is in terms of the ideas that now form the mainstay of immunological research and whose roots are spawned from the promise of the anti-idiotype concept. Among the applications of the INT is understanding the structural implications of the antibody-mediated network that has the potential for innovation in terms of rational design of reagents with biological, chemical, and pharmaceutical applications that underlies concepts of reverse immunology which is highlighted herein.

## INTRODUCTION

Scientific concepts can spring up now and then that captures the attention of scientific thought, only to be replaced by new ideas (Bornholdt et al., 2011). However, some concepts remain latent for years waiting to be rediscovered. Interestingly, this phenomenon has been modeled mathematically based on cooperative events in the evolution of ideas. The modeling suggests that systems with high innovation rates tend to contain a high degree of noise, along with many small domains of ideas that are constantly generated and replaced. In contrast, systems with low innovation rates tend to have low noise and a state that remains dominant for a long time until a single event replaces it (Bornholdt et al., 2011). Immunology seems to operate on two gears. At the system level, it is of a low innovation type with just a couple of theories slowly rising and/or falling over its lifetime of about a century. At the level of its bordering with molecular biology and cellular physiology, the avalanche of data spurs a much more intense flow of parallel concepts, e.g., mechanisms of antigen receptor repertoire generation, cytokine networks, suppression, lymphocyte population structure, etc. These are concepts that emerge often in loose relationship to each other as they address different domains of the immune system. Although of local importance, they often become fashionable and temporarily generalized, attempting to explain more than they can. This is due, at least in part, to the big theories having a hard time catching up because of their slower development. One such concept is the idiotype network theory (INT) brought forth by Jerne (1974), 1984. The INT postulates that a population of antibodies forms a hierarchical and dynamic network of interconnected elements that define the regulation of the immune system. The promise of the anti-id concept lies in (1) elucidating the immunological mechanisms associated with the regulation of the immune response, (2) defining how nature developed its own approach to reverse engineering which is applicable to vaccine design, (3) their use as vaccines and immunotherapeutics, and (4) their utility in understanding self-tolerance and control of lymphocyte homeostasis.

## THE FOUNDATION OF THE STORY

The basis of the INT is the concept of the idiotype (Id). An Id is a shared characteristic between a group of B cells (immunoglobulin) or T cell receptor (TCR) molecules based upon the antigen binding specificity, and therefore structure of their variable region. The variable region of TCRs and immunoglobulins contain complementarity-determining regions (CDR) with unique amino acid structure that determines the antigen specificity of the receptor. The structure formed by the CDR is known as the idiotope. The term Id is often used to describe the collection of multiple idiotopes, and therefore overall antigen binding capacity, possessed by an antibody. Immunoglobulins or TCRs with a shared idiotope are the same Id. The antibody Id is determined by gene rearrangement, junctional diversity, palindromic nucleotides at sites of single-strand breaks, N-nucleotides, and somatic hypermutations. Inherent to the INT is the relationship between the combining site (paratope) for antigen and the expression of an Id (idiotope).

**FIGURE 1:**
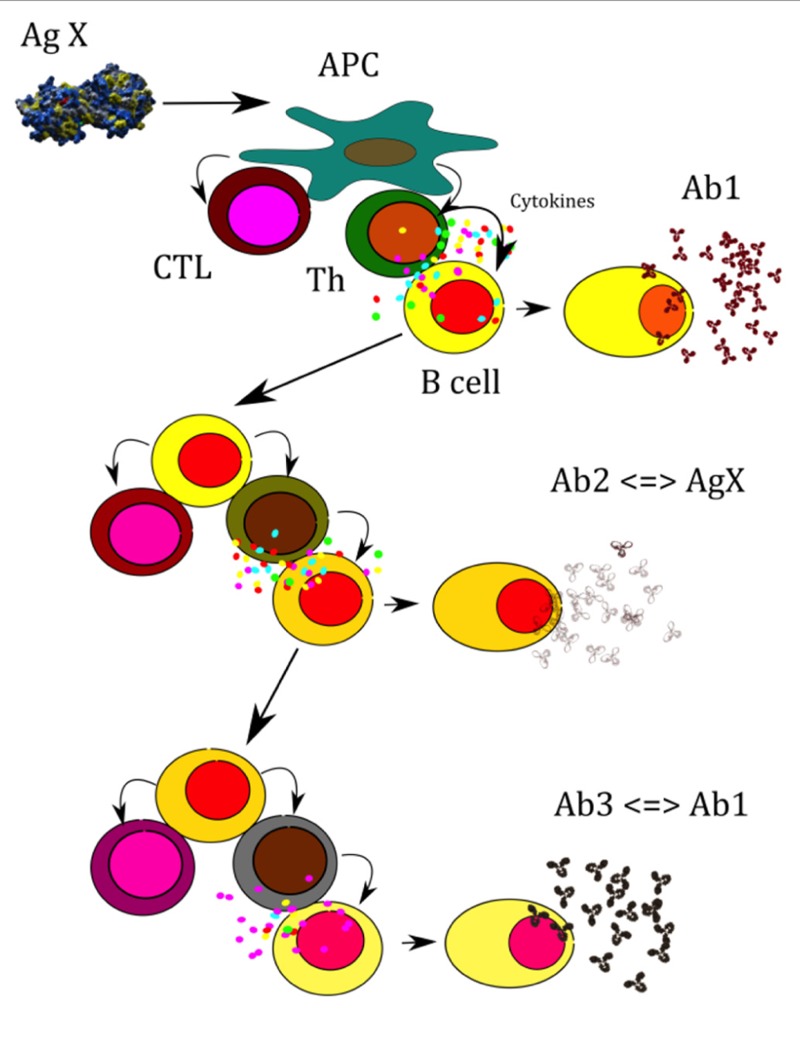
**A model for how conventional T–B collaboration could represent a cellular mechanism by which complementary Id^+^ (Ab1), anti-Id (Ab2), and anti-anti-Id (Ab3) antibodies communicate in a T-cell-dependent manner**. A central tenet of the network theory is that the interaction between Id^+^ and anti-Id immunoglobulin has regulatory consequences in addition to generating cross-reactive immune responses to external antigens. The unification of these ideas is illustrated as adopted from a model presented by (Uner and Galvalchin, 2006). An antigenic determinate X is processed and presented by an antigen-presenting cell (APC) in association with major histocompatibility complex (MHC) class II molecules to an antigen-specific T- helper cell or, in association with class I MHC, to an antigen-specific cytotoxic T cell. Signals from the T-helper cell lead to the activation of B cells that recognize on their own an epitope on X and produce anti-determinate X reactive antibody (Ab1). Anti-X producing B cells, as APCs, present peptides of anti-X (idiotypic Y peptide) in association with class II to idiotype Y-reactive or specific T-helper cell and, in association with class I, to idiotype Y-specific cytotoxic T cells. Signals from the activated idiotype Y- reactive T cells lead to the activation of anti-idiotope Y-producing B cells (Ab2). Likewise, in this context extracellular Id^+^ immunoglublin (Ab1) is endocytosed and processed by APC, resulting in Id-peptides that are presented on MHC Class molecules to Id-specific CD4^+^ T cells. Anti-Y producing B cells, as APCs, present idiotypic Y peptide in association with Class II to idiotype Z-reactive or specific T-helper cell and, in association with Class I, to idiotype Z-specific cytotoxic T cells. Signals from the activated idiotype Z-reactive T cells lead to the activation of anti-idiotope Z-producing B cells (Ab3).

The Network Theory of Jerne postulates that the immune system functions as a regulatory network that is comprised of Ids (Ab1s) and their anti-Ids (Ab2s) in which B cells and other antigen-presenting cells (APC) provide for antigen processing (**Figure 1**). The inherent relationships of the network hierarchy activate both B and T cells through idiotypic network determinants that mimic the three-dimensional structure of the nominal antigen, and thereby activate Ab1 precursors reactive with foreign or self-antigens. They may also be responsible for the stimulation and maintenance of memory T lymphocytes. Thus, MHC-restricted T cells appear to recognize immunoglobulin by the same rules as those that apply to recognition of proteins in general. Antibody Ab1, synthesized in response to a primary antigen, in turn elicits a secondary antibody Ab2. B cell clones recognizing idiotopes on Ab1 in the generation of Ab2s are a heterogeneous population displaying multiple specificities. Sometimes, immunization with Ab2 induces antibodies (Ab3s), which resemble Ab1s as induced by the original or nominal antigen.

**FIGURE 2:**
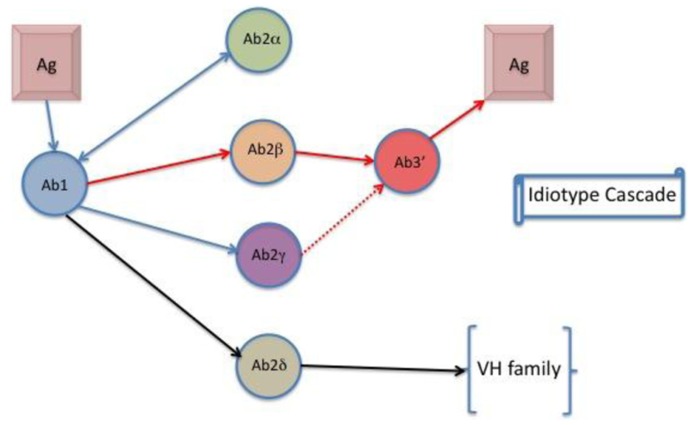
**The idiotype cascade**. Ab1 binds to anti-idiotypic antibodies in ways associated with Ab2 properties. Ab2δ is a new designation that defines binding to framework regions of Ab2 associated with the Ab2 VH family.

This model suggests that conventional T–B collaboration can explain communication between complementary Id^+^ and anti-Id antibody at the cellular level that integrates present and previous data on B cell regulation by Id-specific T cells (Jacobsen et al., 2010). Ab2 antibodies with antigenic properties have been recognized as two types: one that is the “internal image” binding to the antigen binding CDR, and another that binds close to the antigen binding Ab1 site. The first Ab2 termed by Jerne as Ab2 beta, the later as Ab2 gamma (Kohler et al., 1989). Ab2 alpha is defined as an anti-Id without internal imagery to the native antigen. A fourth kind of anti-Id has been described that binds to a framework region of VH (variable region heavy chain) families (Muller et al., 1991; Wang et al., 1995). This Ab2 is classified as Ab2 delta (Kohler, unpublished data). The role and potential of Ab2 delta in vaccine development is currently being explored. Ab2 alpha or Ab2 delta may exhibit a regulatory effect on the production of antibody bearing the Ab1 Id. **Figure 2** shows a flow diagram of idiotypic interactions and **Table 1** lists the different Ab2s.

**Table 1:** Nomenclature of Ab2s.

Anti-Id class	Property	Reference
Ab2 alpha	Non-binding site idiotope	Jerne (1974)
Ab2 beta	Internal antigen image idiotope	Jerne (1974)
Ab2 gamma	Near antigen binding site idiotope	Kohler et al. (1989)
Ab2 delta	Non-binding site, VH-specific and outbred shared idiotope	Kohler, unpublished

The INT is perceived to have had better days and is considered to be a case in the rise and fall of a scientific paradigm in today’s mainstay of immunological research (Eichmann, 2008). What initially appeared as an exciting new perspective of the immune system is now viewed as a scientific vagary, and is perceived to be largely abandoned (Eichmann, 2008). Literature searches of select keywords over the last three decades highlight a decline in publications in the respective topic areas except for anti-Id vaccines (**Table 2**). Yet it is this promise that has drawn skepticism, but remains exciting for some because anti-Ids continue to prove their potential in the clinic (; Maruyama et al., 2000; Li et al., 2002; Ruffini et al., 2005; Lee et al., 2007; Neninger et al., 2007; Ai et al., 2009; Fernandez et al., 2010; Hernandez et al., 2011; Inoges et al., 2011; Ng et al., 2012).

**Table 2:** Distribution of publications based upon keywords associated with the network theory.

PubMed keyword search	[“1980/01/01” (date – publication): “1990/12/31” (date – publication)]	[“1991/01/01” (date – publication): “2000/12/31” (date –publication)]	[“2001/01/01” (date – publication): “2012/8/28” (date – publication)]
Idiotypic network	254	249	105
Idiotypic network theory	32	19	17
Anti-idiotype antibodies	4229	3326	2794
Anti-idiotypes	180	100	29
Anti-idiotypic vaccine	169	268	292
Idiotype	3432	1596	758
Regulatory idiotype	411	65	40
Idiotope	262	158	27
Idiotypy	62	14	2

Perhaps the main weakness of the INT is its claim for generality while including a very restricted set of components and interactions in the immune system. With the buildup of data on its complexity, processes, and properties therein like memory, tolerance, and repertoire selection found better explanations in a less exciting, reductionist context. This made the speculations of the INT role in these processes at best overstated. Did it lose credibility altogether? No – only its level of generalization was corrected. So the “innovation” that brought about the paradigm shift did not replace the theory in its domain, but rather, expanded the field showing that there is much more to immune mechanisms than antibodies, B cell clones and their interactions. This is a promise fulfilled.

There are two aspects of the INT that not only survived its fall but even saw a major development becoming its most significant legacy. The first, is the role of the network in immune regulation as an important participant in the assortment of an improved clinical outcome (Abdeen, 2011). This role has been confirmed by the finding that “auto-anti-idiotypic” antibodies against the induced antibody (Ab1) arise during the immune response in mice (Kluskens and Kohler, 1974; Cosenza, 1976) and in humans (Stefanescu et al., 1993). The second, fundamentally proposes that antibodies can themselves function as surrogates of antigens and immunogens (McNamara et al., 1984; Hernandez et al., 2008, 2011) that redefines anti-Ids as network antigens (Kohler et al., 1989). In short, a promise that has wide sweeping implications is that anti-Ids can function as mimics of ligands and antigens, functioning as surrogates binding competitively to antigen-specific cell receptors. Thus, intrinsically, any antibody can function as a ligand surrogate by its very nature. This realization defines anti-receptor antibodies as surrogate ligands and indicates that this promise of the INT is still in the mainstream of immunological research but has morphed into the notion of approaches to targeted therapy (Goldenberg and Sharkey, 2012).

A major theoretical contribution is also the reassessment of the role of antibody polyspecificity. Molecular mimicry is now firmly considered as the basis of many autoimmune disorders where a foreign antigen shares sequence or structural similarities with self-antigens (Chastain and Miller, 2012; Cusick et al., 2012). Molecular mimicry in this context has typically been characterized at an antibody (B cell) or T cell level. Even if an epitope mimic can support a cross-reactive T or B cell response *in vitro*, its ability to induce an autoimmune disease *in vivo* will depend upon several factors including the appropriate presentation of the mimicry of host antigen expressed on the target tissue. In the case of T cell mimics, the ability to mimic an epitope to induce a proliferative response depends upon engagement of the MHC-peptide complex with the TCR (Davies, 1997). However, few investigators truly realize that peptides can be overlapping B cell and T cell epitopes and or simultaneously involved in the interaction with anti-idiotypic B and T cells, behaving as a regulatory idiotope (Perez et al., 2002). On the other hand, the different principles of repertoire selection makes T- and B-cell antigen receptors independent in their polyspecificity (Wucherpfennig et al., 2007) and, generally, only one of the epitopes will be triggering the process of mimicry but will recruit the help of the other because of mimicry. Indeed, the promise of understanding regulation is evident.

Despite that pathogen-associated animal models were often used to validate vaccination with anti-Ids, anti-Id vaccination has made it to the clinic for cancer. A number of monoclonal antibodies that mimic distinct human tumor-associated antigens as well as Id vaccines have demonstrated encouraging results in clinical studies for solid tumors (; Maruyama et al., 2000; Ruffini et al., 2005; Lee et al., 2007; Neninger et al., 2007; Fernandez et al., 2010; Hernandez et al., 2011; Ng et al., 2012). While the theoretical hypothesis is sound, trials have been limited and have not been tested prospectively. Several studies have provided proof-of-principle of biological efficacy of these vaccine types, clinical efficacy, and even clinical benefit in small studies conducted in humans (Bendandi, 2009). However, several randomized clinical trials have failed to achieve their main endpoints for reasons that might be unrelated to a vaccine (Bendandi, 2009; de Cerio and Inoges, 2009). While skepticism toward this type of approach is mounting, better-designed clinical trials might prove to show efficacy for vaccinations that emphasizes idiotypy (Bendandi, 2009; de Cerio and Inoges, 2009). Still, the greatest challenge of immunotherapy by means of antibody-based vaccines, especially in the context of solid tumor therapy, is to identify the antibody that will function as a true surrogate-antigen generating both humoral and cellular immune responses to tumor cells.

It may seem worth mentioning here a recently debated aspect of immunoglobulin immunogenicity. The description of immunodominant regulatory T cell epitopes in the constant part of the immunoglobulin molecule (De Groot et al., 2008; Cousens et al., 2012) seems to add a balancing force to the immunogenic variable region with its T and B cell idiotopes. The net effect, though, should counterbalance the particular idiotopes not with the self-epitope found on the injected Ab2 molecule but with the combined amount of the identical constant region epitopes of the total endogenous immunoglobulin. For that matter, one can actually argue for a role of (and competition with) all immunodominant self-epitopes continuously presented. Obviously, a relatively negligible amount of immunoglobulin injected as an immunogen would hardly change the repertoire of self-epitopes presented and the “Tregitope” concept changes nothing in this case.

## BIOLOGICAL VERSUS CHEMICAL MIMICRY

Lately, emphasis is being placed on using neutralizing antibodies as templates to reverse engineer immunogens by working back to reconstruct the neutralizing epitope by structure-based design technology with the intent to induce neutralizing antibodies by the mimicking immunogen (Sattentau and McMichael, 2010; Bundle et al., 2012; Lipinski et al., 2012). Id/Anti interactions are nature’s own approach to reverse engineering. Antigen binding of antibodies is mediated by atomic interactions within complementary surfaces between antibodies (paratope) and antigens/determinants (epitope). The interaction between antibodies and antigenic determinants is determined not necessarily by a primary chemical structure, but by the stereochemistry of the antigen. Reviews over the years have discussed idiotypic relationships from a structural viewpoint (Kieber-Emmons et al., 1986; Kieber-Emmons et al., 1987a; Poljak, 1994). In most cases, Ids are associated, fully or entirely with the CDR of antibodies indicating that a binding site is both recognizing and being recognized. Antibodies are known to cross-react with non-structurally related molecules, and, paradoxically, the distinction between paratope and idiotope is not straightforward because mimicry is attributed to the CDRs of Ab2s and requires adjustment for the development of a rationale for immunomodulatory approaches using antibodies as immunogens (Kohler et al., 1989). But why should this interplay be structurally determined?

One line of evidence that suggests chemical mimicry can regulate biological mimicry stems from sequence analysis of the anti-Ids F6 and 4C11, which were defined as anti-Ids for phosphorylcholine (PC; Huang et al., 1988). Sequence and structure analysis suggested that the CDR2 of 4C11 was unique, displaying a positive and negative charge distribution that mimics the charge distribution of the PC head group that defines a correlate with function and structure (Kieber-Emmons et al., 1987b; Cheng et al., 1988). In fact peptides that mimicked PC were shown to compete with PC for antigen binding using a reverse engineering approach (Kieber-Emmons et al., 1987b). The crystal structure of anti-idiotypic monoclonal antibody 409.5.3 and its idiotypic Fab fragment complex (a feline infectious peritonitis virus neutralizing antibody) also illustrates the manner in which two Fabs interact by direct placement of their complementary CDRs (Ban et al., 1994). Analysis of the Ab1–Ab2 interface reveals that there is high degree of structural and chemical complementarity between the two as is observed in other antigen-antibody complexes.

Superimposed on structural complementarity though is the idea that antibodies are polyspecific. Polyspecificity is a basis for functional mimicry. Unlike the classical notion in the sense of a signal, switched from the legitimate target to an inappropriate self-target, here the context is rather of converging signals coming from a class of ligands carrying a common biological meaning (Cohn, 2008). For example, a common cross-reactivity between carbohydrates and intracellular hydrophobic determinants, points to a possible biological function of carbohydrate/protein mimicry (unpublished observations). It may overlap structural markers of dangerous change of the internal environment. Anti-Ids that mimic carbohydrate antigens are abound in description. The premise for antigenic mimicry is thought to rely on the presentation of reactive groups such as hydroxyl groups that topologically correspond between the antigen and the anti-Id based on hydrogen bonds, which may or may not be supplemented by hydrophobic interactions.

Stereochemical similarity may determine an immunochemical likeness even between molecules belonging to different classes of compounds, for example, between peptides and polysaccharides (Luo et al., 2000; Cunto-Amesty et al., 2001a; Monzavi-Karbassi et al., 2007). Let us consider an antibody and an antigen it is specific for. Almost always other structures can be found that bind to the antibody, competing for the antigen. When such cross-reactive determinants also induce antibodies cross-reactive with that same antigen, they are defined operationally as mimics of that same antigen. This algorithm for defining mimics does not necessitate complete structural identity between the molecular interfaces in the antibody/antigen and the antibody/mimic complexes. Furthermore, due to the very nature of mimicry as an instance of antibody polyspecificity, it seems intuitively obvious that, in general, different mimics bind to the nominal antibody with diverse footprints. Thus, anti-Ids need not display an exact structural correspondence with the nominal antigen, let alone different stereochemical aspects from which the same epitope can be recognized by different antibodies (Luo et al., 2000; Cunto-Amesty et al., 2001a; James and Tawfik, 2003; Pashov et al., 2005; Monzavi-Karbassi et al., 2007; Talavera et al., 2009). Although this is the general principle, there are anecdotal examples of mimicry with a very high structural fidelity, especially in Id-anti-Id systems, proving that it is not only possible but also of not too low probability.

## STRUCTURAL CHARACTERIZATION OF ANTIBODY RECOGNITION

The three-dimensional structures of several anti-Id antibodies, either alone or in complex with an idiotope have been determined (Garcia et al., 1989; Bentley et al., 1990; Ban et al., 1994, 1996; Evans et al., 1994; Chang et al., 2005). The first insight into the structural aspects of anti-idiotypic antibodies comes from Ab1 (FvD1.3), Ab3 (Fv E5.2) antibodies in the lysozyme system (Fields et al., 1995; Braden et al., 1996). The study showed that the Id-anti-Id interaction involves all six CDRs of each molecule although the interaction between E5.2 HCDR1 and D1.3 is achieved only through bridging water molecules. In the D1.3 complexes with the lysozymes, the conformation of D1.3 LCDR3 in complex with E5.2 is dependent on the electrostatic nature of the residue in contact with the L3 backbone.

The anti-Id antibody mimics the lysozyme by a strong topological similarity in hydrophilic interactions and by making a comparable number of Van der Waals contacts to the combining site of the Ab1. This mimicry is well exemplified by the patterns of hydrogen bonding: six of the 14 protein–protein interface hydrogen bonds in the Ab1-anti-Id Ab2 complex are superimposable with hydrogen bonds in the Ab1-lysozyme interface, suggesting fidelity in hydrogen bonding as a basis for cross-reactivity and mimicry. Not perfect fidelity but close. Interestingly, the solvent structure of the Id-anti-Id antibody complex is observed to contribute to the mimicry of the lysozyme in the context of recognition.

The mimicry of E5.2 for lysozyme does not however extend to the topology of the non-polar surfaces of E5.2 and lysozyme, which are in contact with D1.3 as revealed by a quantitative analysis of the contacting surface similarities between E5.2 and lysozyme. It was concluded that the anti-idiotypic antibody E5.2 mimics lysozyme in its binding interactions with D1.3. Validating these observations, E5.2, used as an immunogen, induces an anti-lysozyme response (Fields et al., 1995). This observation underlies an essential part of the INT; that hydrogen bonding is an important element that provides directionality to defining the degree of fidelity which is often overlooked in deciphering and discussing the structural basis for mimicry.

### SEQUENCE RELATIONSHIPS THAT DEFINE MIMICRY

Bruck et al. (1986) described one of the earliest observations of shared sequence homology between antibodies and antigens. Importantly, this system also taught, in a series of papers, how to develop peptides and small molecules from antibody structure (Williams et al., 1988, 1989, 1990, 1991; Kieber-Emmons et al., 1997). Even before the work of Bruck et al., insight as to the sequence relationships between antigens and antibodies was emerging (Vasta et al., 1984). To estimate the minimal structural requirements for cross-reaction of idiotypic determinants, Vasta et al. (1984) determined the capacity of monoclonal antibodies specific for the Id of the PC-binding myeloma protein TEPC-15 for cross-reactivities with the PC-binding, acute-phase protein C-reactive protein (CRP), and the hemagglutinin from the horseshoe crab *Limulus polyphemus* (limulin), which binds sialic acid and PC. Human CRP displays calcium-dependent binding to a variety of autologous and extrinsic ligands, and aggregates or precipitates, but which binds with highest affinity to PC residues. Neither CRP nor limulin showed significant overall sequence homology to vertebrate immunoglobulins.

However, CRP, limulin, and TEPC-15 VH shared short stretches of homology (8–10 amino acids) that mapped to a stretch comprised of the CDR2 and third framework region of the TEPC-15 VH. These results suggested either evolutionary convergence forced upon molecules of diverse evolutionary histories because of steric requirements of binding the same ligand, or a conservation of primitive combining site gene segments in evolution. Further studies showed that CRP displays the same idiotope as an antibody that shares its specificity for PC (Swanson et al., 1991), whereby the shared epitope on TEPC15 and CRP was composed of similar charged residues. The mechanism by which one molecule has evolved, or was obtained by chance, similar amino acid sequences or the homologous three-dimensional crystal structure of immunodominant epitopes remains a mystery.

### B CELL INTERACTIONS

The *in vivo* and *in vitro* results involving anti-Ids and PC hapten suggest that idiotope antigens can function like nominal antigens to induce antigen-specific B cell responses, providing a mechanistic view for priming and boosting primed B cells (Huang et al., 1986). The mechanisms of thymic-dependent B cell activation induced by idiotope and nominal antigen are similar in that MHC-restricted T and B cells interactions require cognate recognition (Huang et al., 1986). An important take home message was suggested that the combined use of idiotope and nominal antigens in an immunization protocol might provide the maximal protective immunity. This translates the primary observation embedded in anti-Id vaccination protocols into the present day use of diversified prime and boost strategies to enhance anti-tumor immunity (Grosenbach et al., 2001; Monzavi-Karbassi et al., 2003; Nolz and Harty, 2011).

Mimicry is a powerful concept to develop tools for delineation of the mechanisms whereby antigens affect lymphocyte function (Weissberger et al., 1983; Shenk et al., 1984). Antibody specificity is determined by a limited number of residues. This fact has prompted the synthesis of small peptides based on CDR sequences, which retain binding properties and functions of the intact antibody. Studies also suggest that peptides derived from CDRs may act likewise effectors of the innate and adaptive immune response opening a new scenario about their interplay with the cellular immune response (Westerink et al., 1995; Gabrielli et al., 2009).

Because B1 cells can strongly activate T cells and induce T helper type 1 (Th1) cell differentiation in the context of antigen presentation, we have been testing how carbohydrate mimetic peptides (CMPs) mediate T cell responses. We have shown that immunization of mice with a CMP reactive with anti-GD2antibodies (GD2 is a tumor antigen expressed typically on cells of neuronal origin), induce GD2 reactive IgM antibodies (Wondimu et al., 2008). This CMP also induces a DTH response to GD2-positive D142.34 cells, while no response was observed against the GD2-negative expressing cell line B78.H1. The anti-GD2 IgM induced by CMP plays the role of an initiating factor for a DTH response perpetuated by T cells cross-reactive with CMP and an unknown antigen on the tumor cells line, which have been stimulated during the priming with CMP. This observation suggest that the dual character of a CMP carrying a T cell epitope but also mimicking unrelated carbohydrate epitope, provides for long-term IgM responses by promoting other aspects of cooperation between particular B cell subpopulations and CMP-specific T cells (Cunto-Amesty et al., 2003).

We further demonstrated that CMPs direct the generation of tumor-associated carbohydrate antigens (TACA) reactive antibodies in immune deficient *Xid* mice that generally fail to respond to T independent antigens (Cunto-Amesty et al., 2001c). Depending on formulation, CMPs can target repertoire compartments inaccessible to native TACA in these mice. Therefore, we hypothesize that CMPs, peptides derived from anti-Id CDRs and anti-Ids can stimulate B cell compartments and activate effector cells that bridge innate and adaptive immunity (Pashov et al., 2010).

### TARGETING B CELL IDIOTYPES AS A SPECIAL CASE

B cell malignancy is usually derived from a single expanded B cell clone, which expresses an immunoglobulin with a unique Id (Shaffer et al., 2002). Therefore, anti-B cell antibodies targeting Ids are especially useful to probe the biology of B cell malignancies. The use of anti-B cell antibodies targeting Ids is also a model for therapeutic modality targeting receptors because the expression and signaling of the membrane bound immunoglobulin constituting the B cell receptor (BCR) is critical for cell survival and proliferation (Choi and Kipps, 2012; Kenkre and Kahl, 2012). Anti-Ids function as anti-receptor antibodies in this case whereby they directly recognize the tumor-associated Id (antigen) to mediate both antibody-dependent cellular cytotoxicity and signaling-induced cell death (Tutt et al., 1998). Lymphoma has been the model for the clinical utility of “anti-Id” therapy (Hsu et al., 1997; Ruffini et al., 2002), serving as a tumor-specific antigen for therapeutic vaccine development. Immunization of lymphoma patients with their own tumors generates humoral and cellular immune responses to their lymphomas (Neelapu et al., 2006). However, the clinical impact of an Id directed immune response is still under evaluation (Mahaseth et al., 2011) and often reviewed (Bendandi, 2009; Brody et al., 2011; Inoges et al., 2011; Hollander, 2012).

Structurally, tumor-specific Id might be considered in the context of a privileged target for vaccine therapy. The main goal of any biological therapy of tumors is the selectivity of the agent used with immunotherapy representing the protypical approach. In the case of lymphoma, the tumor-specific antigen is the unique variable region of the immunoglobulin produced by the malignant clone and anti-Ids use is based on their ability to detect highly restricted or “private” Ids therein. “Private” Ids are speculated to be associated with CDR regions while so-called “public” Ids might be related to framework residues. Such “private” Ids are reflected in somatic mutation, which might be relatively unique to an individual. Consequently, a major obstacle in production of Id vaccines derives from its patient-specific nature that requires the generation of a custom-made product. Anti-Id/Id interactions are also known to be mediated by framework residues (Brown et al., 1991), suggesting that framework residues affect Id expression (Corti et al., 1994) as originally proposed (Kieber-Emmons and Kohler, 1986; Kieber-Emmons et al., 1987a). However, because the focus is on developing both humoral and cellular immune responses to B cell Ids, CDRs are more likely targets for T cells. T cell lines generated from lymphoma patients actively immunized with Id protein were shown to specifically recognize CDR-derived peptides (Baskar et al., 2004). Synthetic peptides corresponding to hypervariable regions of immunoglobulin heavy chain have been described to be specifically stimulated by CD4^+^ and CD8^+^ T cells to proliferate and secrete proinflammatory cytokines in an MHC-associated manner (Baskar et al., 2004).

However, the plasticity of the BCR repertoire and the structural similarities among BCR and TCR allow antibodies to effectively mimic TCR binding to MHC (Polakova et al., 2000). Because a large number of HLA-binding idiotypic peptides can be identified among antibody hypervariable sequences, such peptides may spontaneously induce a type I MHC class I- as well as class II-restricted memory T cell response (Hansson et al., 2003). Early studies suggested that antigen-binding receptors on T lymphocytes and IgG antibodies with the same antigen-binding specificity as the TCRs display shared or identical Ids (Binz and Wigzell, 1975). Such shared Id might be associated with framework residues. While some reports associate CTL responses to framework residues (Trojan et al., 2000; Gricks and Gribben, 2003) framework peptides might play a more fundamental role in regulation in which Tregs induced by a shared Id epitope can systemically suppress T cell responses against Id-derived and immunodominant foreign epitopes *in vivo* (Warncke et al., 2011).

### T CELL INTERACTIONS

In addition to inducing antibodies, Ids/anti-Ids also induce cellular responses. Such studies suggest that T cells need to be integrated into idiotypic regulation (Jacobsen et al., 2010). The ability to prime T cells derived from normal HLA-matched donors, rather than patients, may have direct application to current strategies, designed to generate allogeneic tumor-specific T cells for adoptive transfer (Weng et al., 2011a,b). MHC-restricted T cells appear to recognize immunoglobulins by the same rules as those that apply to recognition of proteins in general (Eyerman et al., 1996). In this context it is easy to rationalize that an Id^+^ B cell presents Id peptides to Id-specific T cells. It follows that an Id^+^ B cell primarily will be regulated by a limited set of T cells specific for highly expressed germ-line (maybe) Id^-^ and to a lesser extent by a diverse set of T cells specific for a multitude of Id-peptides derived from somatically mutated (maybe) anti-Id Ab. As for the anti-Id B cell, the converse is expected to hold true. Thus, complementary Id^+^ B cells and anti-Id B cells are anticipated to be regulated by partly overlapping sets of Id-specific T cells whose Id-peptide/MHC class II ligands are expressed to different levels by the two complementary B cells (Jacobsen et al., 2010). More importantly, observations that T cells are activated by Id peptides associated within the CDRs imply that T and B cell epitopes do overlap and such peptides function as regulatory (Perez et al., 2002). Id-specific T cell clones can recognize and respond to idiotypic determinants on B cells (Eyerman et al., 1996; Osterroth et al., 2000; Wen et al., 2001). Id-reactive T cells are MHC-restricted and recognize idiotypic determinants in the form of peptide fragments in the context of MHC class II molecules presented on APCs. This type of binding suggests that a conformational Id was processed and presented to T cells in a manner that maintained its structure. It is known for sometime that the overlapping topology of T and B cell epitopes within synthetic peptides does not necessarily impair B cell immunogenicity (Harris et al., 1996).

In terms of autoimmunity, molecular mimicry is defined as the theoretical possibility that sequence similarities between foreign and self-peptides are sufficient to result in the cross-activation of autoreactive T and B cells (Ang et al., 2004). Apart from Ab2s inducing antibodies, there is evidence for differences among the Ab1-Ab2-Ab3 cascade induced by protective and non-protective anti-Id attributed to cellular responses (Raychaudhuri et al., 1990). Among various anti-Ids typed serologically as an internal image Ab2 of the mouse mammary tumor virus tumor-associated antigen gp52, only one induced protective immunity and was effective in immunotherapy. The DNA sequence of the variable regions of six anti-Ids was determined. Search for amino acid sequence homologies between the Ab2s and gp52 showed the strongest similarities in sequence in the CDR2 of the light chain for the protective Ab2 with a T cell epitope on gp52. This finding was the first to raise the question of where the short peptides, which carry T cell-defined epitopes, are located and their relationship with the tumor antigen.

In more recent studies, Ab2s with known amino acid sequence displayed similarity with peptides from a corresponding tumor antigen (carcinoembryonic antigen, CD55, and human high molecular weight melanoma-associated antigen), but differed from the tumor antigen peptides by the presence of side chains known to mediate stronger binding with MHC (Spendlove et al., 2000; Kawano et al., 2005; Ullenhag et al., 2008). In particular in the CD55 system amino acid homology was identified between three CDRs of the anti-id and three regions of CD55 (Spendlove et al., 2000). Anti-anti-idiotypic (Ab3) polyclonal antibodies raised against the Ab2 showed specific binding to these peptides. The antibodies were also found to bind synergistically to combinations of these peptides, indicating cooperatively between the peptides in stabilizing antibody binding (Spendlove et al., 2000). These findings contribute to identifying the mechanism by which a human anti-idiotypic antibody is able to mimic a tumor-associated antigen and stimulate anti-tumor B and T cell responses.

A more direct approach is proposed to use anti-Ids and monoclonals to target antigens directly to APC (Durrant et al., 2011). One approach entering the clinic stimulates anti-tumor immunity using monoclonals genetically engineered to express tumor-specific T cell epitopes to enhance T cell activation to eradicate tumors (Durrant et al., 2011). This work is an off-shoot of early ideas on antigenizing antibodies (Zanetti, 1992). However, natural regulatory T cells might control the specificity of T cell-mediated anti-Id immunity (Warncke et al., 2011). Tregs induced by a shared Id epitope can systemically suppress T cell responses against Id-derived and immunodominant foreign epitopes *in vivo* (Warncke et al., 2011). Collectively, these results further highlight the promiscuity of peptide sequences were a single antibody or TCR can be activated by a few crucial residues (Polakova et al., 2000). Consequently, again choosing the correct anti-Id is a challenge.

## STRUCTURAL CONSIDERATIONS IN THE DESIGN OF MIMICS

In 1986, we suggested that the ultimate goal for Id vaccines was to prepare peptide vaccines derived from idiotypic sequence regions mimicking antigenic structures (Kieber-Emmons et al., 1986). We have accomplished this in an infectious model, being the first to do so (Westerink et al., 1995) and defined many of the paradigms associated with using such peptides (Cunto-Amesty et al., 2001b). We have applied lessons learned from the network theory to develop peptides that mimic TACA (Monzavi-Karbassi et al., 2007), bringing one of them into the clinic in a phase I safety study in breast cancer subjects (Monzavi-Karbassi et al., 2007) and now moving into a phase II trial of high risk breast cancer subjects to prevent recurrence of breast cancer.

Much like anti-Ids, peptide mimics may elicit anti-polysaccharide responses, but fail to elicit the Ids and isotypes observed in the protective response to the microbial antigen (Harris et al., 2002). Functional antibodies depend not only on the host’s ability to mount an immune response, but also on its ability to mount the correct immune response. Whether an antibody response is protective or not depends on both the fine antigenic specificity that may be associated with particular Ids and epitope binding characteristics, and the isotype, determining antibody effector function. And herein lies the problem with mimics; the immune response is only assayed after a choice is made as to which mimic is to be followed. So what lessons can be learned about choosing the correct mimic?

In the first instance, the judicious choice of peptides for testing antibody responses against should be based on the peptide interaction with both the heavy and light chain in order to induce antibodies with similar antigen-specific properties (Luo et al., 2000); as the combination of heavy and light chains will influence specificity (Kabat and Wu, 1991). Thus, both the variable and the constant region of the antibodies induced by a peptide mimic or mimotope must be considered when assessing the success of any immunization. One way to determine this is to use structural information of the antibody–antigen interactions, e.g., reverse engineering concepts.

### FIDELITY OF MIMICRY

We have previously reviewed the structural concepts and approaches used in vaccine design applications that illustrate the value and limitations of using chemical (peptide libraries which are mimics of a ligand) and immunological information to define novel peptide immunogens that function as mimotopes to generate immune responses targeting TACA (Pashov et al., 2005) and glycans on the human immunodeficiency virus (Pashov et al., 2007). In this context we showed early on that concepts associated with pharmacophore design (now considered reverse engineering) could be used to define CMPs applied to vaccine design (Luo et al., 2000; Cunto-Amesty et al., 2001a). We demonstrated that a structure-assisted vaccine design approach, whereby small molecules, defined in crystallographic databases, could be used to theoretically define peptide mimetics emulating the three-dimensional interaction scheme of a native carbohydrate antigen (Luo et al., 2000; Cunto-Amesty et al., 2001a). More importantly it was shown that virtual screening led to motifs being observed experimentally (Luo et al., 2000). We have also shown that by using this approach, an immunogenic peptide can be designed *de novo* (Cunto-Amesty et al., 2001a) and have shown that CMPs reactive with lectins and antibodies can induce antibodies with the same functionality as lectins and antibodies (Monzavi-Karbassi et al., 2005).

To generate sustained immunity to TACAs, we have developed immunogens based on CMPs – a strategy whose clinical promise is supported by our preclinical studies (Monzavi-Karbassi et al., 2007). CMPs can induce anti-tumor cellular responses, including CMP- and TACA-reactive Th1 CD4^+^, and tumor-specific CD8^+^ cells that may compensate for low-titer humoral responses (Monzavi-Karbassi et al., 2001, 2004). Most of all, unlike TACAs, CMPs can prime for memory responses to TACAs (Monzavi-Karbassi et al., 2003), suggesting that the CMPs facilitate cognate interactions between B cells and T cells, which is something that TACAs do not facilitate, but anti-idiotypic antibodies and peptides should and can do.

The question remains of how to enhance the ability of TACA mimetic peptides to induce TACA-specific antibodies with higher titers and association constants. We tested the hypothesis that improving the hydrogen bond pattern through amino acid substitutions in a CMP, to be coincident with that for the carbohydrate ligand, will enhance the ability of CMPs to elicit anti-TACA antibodies with high titers and association constants. Based on anti-Id/Id crystal structures, highly directional bonds represent an important set of interactions to establish a basis for mimicry because they mainly confer the specificity in binding of the peptide and the carbohydrate antigen.

### MIMICS FOR GD2 ANTIGEN

In previous studies, we made use of the crystal structure of the Fab fragment of ME36.1 has been determined (Pichla et al., 1997), showing that its CDRs form a groove-shaped binding site. Molecular modeling has placed a four-residue sugar, representative of GD2, in the antigen-binding site showing much of the interaction with GD2 contributed by heavy chain interaction. Based upon hydrogen bonding schemes with the GD2 antigen, we used conformational and energy analysis to define potential binding modes of a CMP in the crystallographically defined ME36.1 binding pocket (Monzavi-Karbassi et al., 2007). Molecular modeling of the CMP in the ME36.1 binding site indicates that the CMP only shared two hydrogen bonds with the GD2 antigen when binding to ME36.1. This is in contrast to the seven hydrogen bonds formed between GD2 and the monoclonal antibody ME36.1.

Based upon hydrogen bonding schemes with the GD2 antigen, we wanted to determine if we could modify this CMP to increase the level of GD2 antigenic mimicry. Based upon conformational studies we surmised that removing the first three residues of the CMP would result in a peptide with a binding mode with ME36.1 with an increased number of hydrogen bonds in common with the way the GD2 antigen binds to ME36.1 (Monzavi-Karbassi et al., 2007). The redesigned CMP shared five hydrogen bonds in common with GD2 in binding to ME36.1. Computer-based binding studies indicate that the topographical binding mode of this redesigned CMP overlaps that of GD2 in the ME36.1 combining site. Our studies indicate that the redesigned peptide represents a more faithful mimic of ganglioside binding the monoclonal antibody ME36.1 than its original homolog based upon hydrogen bonding of ME36.1 to GD2. Immunization with this redesigned peptide resulted in enhanced antibody responses to GD2 and to tumor cells expressing GD2.

Although structural analysis may raise the confidence that the isolated peptide will have functional value, the induction of cross-reactive immune responses remains the ultimate proof of mimicry. To test if the increase in the level of GD2 mimicry translates into an improved GD2 reactive response, mice were immunized twice with versions of the CMP peptide (P10 original: GVVWRYTAPVHLGDG; P10s WRYTAPVHLGDG; synthesized as MAPs) and then bled 7 days after the boost (Monzavi-Karbassi et al., 2007). Immunization with MAP-P10s induced serum IgM antibodies superior in GD2 binding than serum antibodies induced by P10 (Monzavi-Karbassi et al., 2007). Serum IgM antibodies were also more reactive with the GD2-positive human WM793 cells, suggesting that an improved level of GD2 mimicry lends to an improved antibody response against GD2 (Monzavi-Karbassi et al., 2007). These results validated the hypothesis that mimetics can be more faithful in their mimicking potential. Such results confirm that stereochemically peptides and carbohydrates can bind to the same antibody-binding site, and that peptides can structurally mimic salient features of carbohydrate epitopes binding to a receptor fulfilling a promise of the anti-Id concept with these CMPs being tested in phase I trials.

## SUMMARY

The regulation of immune responses is still in the mainstream of immunology research. However, the paradox of today’s immunology is the lack of correspondence between the progress in basic science and the success of clinical applications. Many clinical trials in cancer currently ongoing aim to either stimulate an anti-tumor immune response or thwart immune suppression. Among those clinical applications are immunotherapeutics that make use of antibodies in some way. Moreover, antibodies and B cells are still considered beyond their effector roles, in terms of regulation and control of the mechanisms of tolerance.

Far from being refuted, concepts derived from INT have the potential to fuel new ideas and therapeutic approaches. Recent studies, reviewed here, confirm that idiotypy concepts hold promise in several aspects. First, although the basic immune phenomena are now known to have their origins in complex molecular and cellular interactions, idiotypic control exists and has undoubtedly its place in the overall immune dynamics, especially in selection of the antigen receptor repertoires. Second, studying idiotypic phenomena unveiled a mechanism of immune system’s natural “reverse engineering” of antigens. A stimulating exercise in system immunology, INT provides also intellectual tools to understand how the immune system preserves structural information. Third, as an ultimate proof of validity, these concepts of decoupling of structural information from its carrier lead to applications in the development of vaccines and immunotherapeutics. Finally, INT helps understand the difference between molecular interactions of variable specificity and immune recognition as a function of the entire system. Thus, it contributes to the construction of an essential immunological paradigm.

On the one hand, the INT tackles the idea that immunoglobulin/immunoglobulin recognition mechanisms play a role in self-tolerance and control lymphocyte homeostasis. Although we know now that this is not the main mechanism of tolerance, signals, generated in the process, clearly control lymphocyte differentiation and homeostasis. Idiotypic interactions are known to participate in B cell repertoire selection, at least early in life and in restricted B cell compartments (Elliott and Kearney, 1992; Dietrich et al., 1993), contributing to dominant self-tolerance. On the other hand, the INT defines a paradigm of surrogate ligands and by extension – mimic-based immunogens. In principle, the level of innovation in INT is actually high, spawning some “high turnover” ideas and some others that form the mainstay of immunological research.

The INT might have been too innovative. Probably it came too much ahead of its time, followed by a heap of new data on structures and mechanisms in immunology that greatly expanded our view. The latter served for a kind of reductionist revenge instead of reassessment and development of the idea (except for isolated attempts, e.g., Varela and Coutinho, 1991). The skepticism in INT utility, that thus ensued, was the largest factor in its rise and fall. An interesting example is the perceived role of diverse T cell subpopulations in anti-cancer responses – a major shift of the emphasis, despite monoclonal antibodies being at the front line of some cancer therapies. Perhaps antibodies have not failed, but we do not know how to appropriately apply them and have not fully grasped the lessons learned from them. That very skepticism in INT is to blame, at least, to some extent for this situation. More importantly, only few appreciated the role of mimicry of antibodies in forming the idiotypic network (Varela and Coutinho, 1991; Coutinho, 1995). This may have contributed also to the premature fall of this beautifully speculative concept. Not unlike suppressor T cells which came back as Tregs, we anticipate that the rising interest in systems biology sooner or later will lead to a reassessment of the role of antigen repertoire networks at the systems biology level.

## Conflict of Interest Statement

The authors declare that the research was conducted in the absence of any commercial or financial relationships that could be construed as a potential conflict of interest.
